# Outbreak of Cryptosporidiosis Among Veterinary Medicine Students — Philadelphia, Pennsylvania, February 2015

**DOI:** 10.15585/mmwr.mm6428a7

**Published:** 2015-07-24

**Authors:** Lauren N. Drinkard, Ashlee Halbritter, Giang T. Nguyen, Patricia L. Sertich, Max King, Sallyann Bowman, Rebecca Huxta, Mary Guagenti

**Affiliations:** 1 University of Pennsylvania

On February 20, 2015, a northeastern university’s student health center was notified of five veterinary medicine students with gastrointestinal symptoms. An investigation was conducted to establish the existence of an outbreak, determine the etiology, evaluate risk factors, and recommend control measures.

All five students had attended a training session at the university’s bovine obstetrics laboratory on February 13, which included the handling of two euthanized calves. Patient symptoms, date of onset, and history of calf exposure suggested cryptosporidiosis. Infection with *Cryptosporidium*, a protozoa that causes watery diarrhea and is transmitted by infectious oocysts via the fecal-oral route (1), is common among calves (2). Symptoms in humans typically begin 7 days (range = 2–10 days) after infection and include intermittent abdominal cramps, nausea, vomiting, fever, and weight loss, lasting approximately 1–2 weeks (3).

Two calves used in the training sessions had been euthanized and frozen at −1.4°F (−17.0°C) on February 11. Approximately 28 hours later, the calves were thawed and detergent-washed by laboratory staff in accordance with standard protocols. Necropsies were performed on both animals on February 23, and revealed *Cryptosporidium* oocysts on an acid-fast stain of an intestine smear from one of the calves.

Interviews revealed that 22 students had attended the training session. Sixteen students reported symptoms, including diarrhea (13 students), abdominal cramps (13), nausea (12), fatigue (eight), vomiting (seven), anorexia (five), headache (four), and chills or sweats (four), lasting 2–10 days. Among the 16 symptomatic students, the median age was 25 years (range = 24–30 years), and 13 were female.

Four symptomatic students submitted stool specimens. One case was confirmed by detection of *Cryptosporidium* oocysts using direct fluorescent antibody testing; the other 15 were classified as probable cases, based on CDC case definitions (1). To account for the possibility of other infectious etiologies, stool specimens were also tested for *Giardia, Cyclospora cayetanensis, Isospora, Salmonella, Shigella, Escherichia coli,* and *Campylobacter*; all tests were negative. The positive acid-fast stain from one of the calves and one of the students with a confirmed case implicated the obstetrics laboratory as the source of the outbreak.

The bovine obstetrics laboratory personal protective equipment (PPE) protocol includes donning of gloves and coveralls before animal handling and cleaning boots and doffing of gloves and coveralls after animal handling, followed by 30 seconds of hand washing with warm water and soap. Face protection is not included in PPE protocols for this laboratory. Although all of the 22 students wore gloves during the training session, the number of students who removed their coveralls or washed their hands afterwards is unknown. At least four of the symptomatic students reported that they did not immediately doff their coveralls.

Cryptosporidiosis outbreaks have been reported among veterinary students (4), usually through contact with infected calves, and are associated with lapses in hygiene (5). In this outbreak, students were infected through contact with euthanized calves that had been frozen and thawed before the training session. *Cryptosporidium* oocysts can survive various environmental pressures, including extended exposures at temperatures as low as −7.6°F (−22.0°C) for >700 hours (6). This cluster highlights the importance of appropriate hygiene and proper animal cadaver handling. Since the likelihood of calves being infected with cryptosporidiosis is high, veterinary medical institutions should ensure that recommendations for PPE and proper hygiene techniques for students and staff are fully implemented.
